# Bump2Baby and Me: protocol for a randomised trial of mHealth coaching for healthy gestational weight gain and improved postnatal outcomes in high-risk women and their children

**DOI:** 10.1186/s13063-021-05892-4

**Published:** 2021-12-28

**Authors:** Sharleen L. O’Reilly, Christy Burden, Cristina Campoy, Fionnuala M. McAuliffe, Helena Teede, Jesper Andresen, Karen J. Campbell, Aisling A. Geraghty, Cheryce L. Harrison, Rachel Laws, Jane E. Norman, Helle T. Maindal, Karsten Vrangbæk, Ricardo Segurado, Vincent L. Versace, Timothy C. Skinner, on behalf of the IMPACT DIABETES B2B Collaboration Group

**Affiliations:** 1 School of Agriculture and Food Science, University College Dublin, Belfield, Dublin, Ireland; 2 UCD Perinatal Research Centre, School of Medicine, University College Dublin, National Maternity Hospital, Dublin, Ireland; 3 Faculty of Health Sciences, University of Bristol, Bristol, UK; 4 Department of Paediatrics, School of Medicine, University of Granada, Granada, Spain; 5 Monash Centre for Health Research and Implementation, School of Public Health and Preventive Medicine, Monash University, Clayton, Melbourne, Victoria Australia; 6 LIVA Healthcare, Copenhagen, Denmark; 7 Institute for Physical Activity and Nutrition (IPAN), School of Exercise and Nutrition Sciences, Deakin University Geelong, Geelong, Victoria Australia; 8 Department of Public Health, Section for Health Promotion and Health Services, Aarhus University, Aarhus, Denmark; 9 Department of Public Health, Center for Health Economics and Policy, University of Copenhagen, Copenhagen, Denmark; 10 School of Public Health, Physiotherapy, and Sports Science, University College Dublin, Belfield, Dublin, Ireland; 11 Deakin Rural Health, School of Medicine, Faculty of Health, Deakin University, Geelong, Victoria Australia; 12 Institut for Psykologi, Center for Sundhed of Samfund, Københavns Universitet, Øster Farimagsgade, København K, Denmark; 13 University Department of Rural Health, La Trobe University, Bendigo, Australia

**Keywords:** Pregnancy, Gestational diabetes, Obesity, mHealth, Implementation, Postpartum, Health coaching, Maternal health, Weight management, Foetal programming

## Abstract

**Background:**

Gestational diabetes (GDM) impacts 8–18% of pregnancies and greatly increases both maternal and child risk of developing non-communicable diseases such as type 2 diabetes and obesity. Whilst lifestyle interventions in pregnancy and postpartum reduce this risk, a research translation gap remains around delivering implementable interventions with adequate population penetration and participation. Impact Diabetes Bump2Baby is an implementation project of an evidence-based system of care for the prevention of overweight and obesity. Bump2Baby and Me is the multicentre randomised controlled trial investigating the effectiveness of a mHealth coaching programme in pregnancy and postpartum for women at high risk of developing GDM.

**Methods:**

Eight hundred women will be recruited in early pregnancy from 4 clinical sites within Ireland, the UK, Spain, and Australia. Women will be screened for eligibility using the validated Monash GDM screening tool. Participants will be enrolled from 12 to 24 weeks’ gestation and randomised on a 1:1 basis into the intervention or control arm. Alongside usual care, the intervention involves mHealth coaching via a smartphone application, which uses a combination of synchronous and asynchronous video and text messaging, and allows for personalised support and goal setting with a trained health coach. The control arm receives usual care. All women and their children will be followed from early pregnancy until 12 months postpartum. The primary outcome will be a difference in maternal body mass index (BMI) of 0.8 kg/m^2^ at 12 months postpartum. Secondary maternal and infant outcomes include the development of GDM, gestational weight gain, pregnancy outcomes, improvements in diet, physical activity, sleep, and neonatal weight and infant growth patterns. The 5-year project is funded by the EU Commission Horizon 2020 and the Australian National Health and Medical Research Council. Ethical approval has been received.

**Discussion:**

Previous interventions have not moved beyond tightly controlled efficacy trials into routine service delivery. This project aims to provide evidence-based, sustainable support that could be incorporated into usual care for women during pregnancy and postpartum. This study will contribute evidence to inform the early prevention of non-communicable diseases like obesity and diabetes in mothers and the next generation.

**Trial registration:**

Australian New Zealand Clinical Trials Registry ACTRN12620001240932. Registered on 19 November 2020

**Supplementary Information:**

The online version contains supplementary material available at 10.1186/s13063-021-05892-4.

## Background

Gestational diabetes (GDM), defined as high blood glucose levels during pregnancy, is an increasing health problem affecting up to 13% of pregnancies worldwide and approximately 17 million births annually [1]. Women with a prior history of GDM are almost 10 times more likely to develop type 2 diabetes (T2DM) over the next 10 years [2]. Excess weight is a known risk factor for T2DM; however, excess gestational weight gained during pregnancy is frequently not lost postpartum due to the many demands on a new mother. This leads to an increased risk of overweight and obesity, GDM in future pregnancies, and T2DM later in adult life [3]. This risk is not confined to just the mother. Early foetal programming means that children born to women with obesity during pregnancy also have higher risks of developing childhood overweight and obesity [4–6]. A multicomponent, life course approach with a focus on prevention and early detection is required to break this cycle and reduce the population burden of diabetes [7].

Strong evidence shows that lifestyle change, with improvements in diet and physical activity level, can reduce the development of T2DM in people at risk [8, 9]. Lifestyle interventions during pregnancy demonstrate optimised gestational weight gain (GWG) with an associated reduction in the risk of GDM [10, 11]. Furthermore, a meta-analysis and behaviour change taxonomy analysis showed the value of interventions with components that target diet, physical activity, and behaviour [12–14]. Pregnancy is a key time to initiate engagement as women are involved with health services and trusted health care professionals during this period. However, it has been well documented that during the postpartum period, women experience a lack of professional support and continuity of care [15]. This can result in significant challenges in terms of engaging women in lifestyle interventions, particularly without prior engagement in pregnancy. As such, the perinatal period represents an important opportunity for developing lifestyle changes during pregnancy and ensuring these are maintained after birth. Whilst lifestyle modifications have proven effective [16–18], there remains a large research translation gap around achieving implementable interventions with adequate population penetration and participation. Effective preventative interventions to date have not moved beyond the research phase to consider implementation into routine service delivery, and novel strategies are required [19, 20].

Smartphones have high-level penetration across socioeconomic groups and provide a convenient and accessible method through which to engage and motivate women to improve lifestyle behaviours. Almost 90% of people in the UK and Spain own a smartphone with similarly high penetrations reported for other EU countries and Australia [21]. In addition, mothers aged 18–49 years spend an estimated 21 h per week on their smartphones [22]. Apps and mHealth can provide ‘around the clock’ information, as well as tailored support at low cost [23]. Recent evaluations of mHealth apps available in pregnancy revealed a large number but few were high quality. They typically used a very small range of behaviour change techniques, and the evidence-based nutrition and physical activity information provided was equally limited [24, 25]. Women with low socioeconomic status commonly use apps during pregnancy but not postpartum because of the lack of quality apps [26]. This highlights the opportunity for intervention delivery through a high-quality, evidence-based app to provide maternal support during pregnancy and postpartum. This mode of delivery could facilitate an affordable intervention with reach and scale to overcome penetration and participation challenges as well as enable tailored support to be delivered on a large scale.

The Bump2Baby and Me study is a multicentre randomised controlled trial (RCT) that aims to investigate the implementation and effectiveness of an mHealth-supported behavioural change coaching programme for women who are at high risk of developing GDM. This RCT is central to an international project designed to demonstrate the real-world implementation of an evidence-based, effective system of care for the prevention of diabetes, overweight, and obesity when delivered across antenatal settings (Impact Diabetes Bump2Baby). The Bump2Baby and Me RCT integrates best practice evidence from previous RCTs that successfully reduced excess weight gain to inform personalised health coaching which is delivered via a smartphone application [16, 17, 27]. This RCT proposes to bridge the health service gap from pregnancy to postpartum using a low-resource, precision medicine, evidence-based intervention. This study aims to evaluate the implementation and effectiveness of the mHealth coaching programme to improve appropriate maternal weight management during pregnancy and postpartum and impact on the growth of the baby, which will contribute to early prevention of maternal and child diabetes, overweight, obesity, and other non-communicable diseases.

## Methods

### Study design and setting

**Fig. 1:**
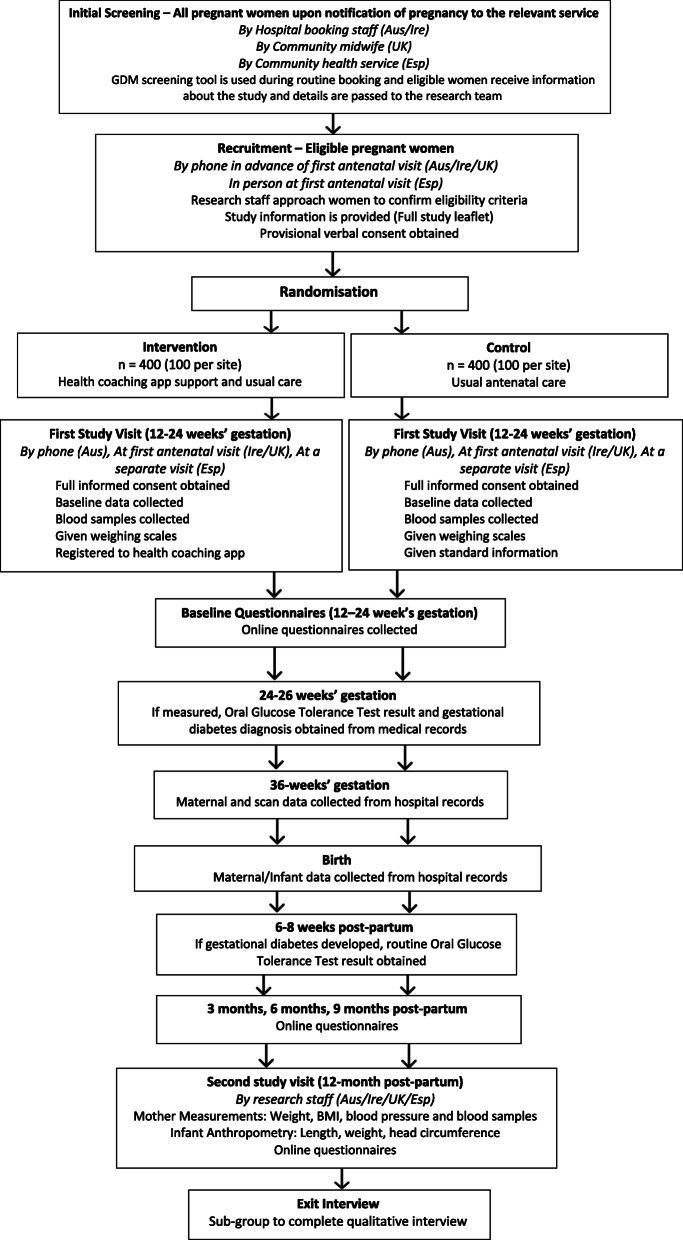
Flow chart with an overview of the time points and data collected in the Bump2Baby and Me study

Bump2Baby and Me is a multicentre single-blind randomised controlled trial involving 800 pregnant women to investigate an mHealth coaching programme for those who are at high risk of developing GDM. This study involves five hospital sites across four countries: National Maternity Hospital in Dublin, Ireland; Clinical University Hospital San Cecilio and Mother-Infant University Hospital in Granada, Spain; Southmead North Bristol NHS Trust in Bristol, England; and Monash Medical Centre in Melbourne, Australia. Ethical approval has been granted for all study sites. This RCT was registered prospectively on the Australian New Zealand Clinical Trials Registry, trial registration number ACTRN12620001240932, on 19 November 2020. The study flow design is described in Fig. 1.

**Fig. 2:**
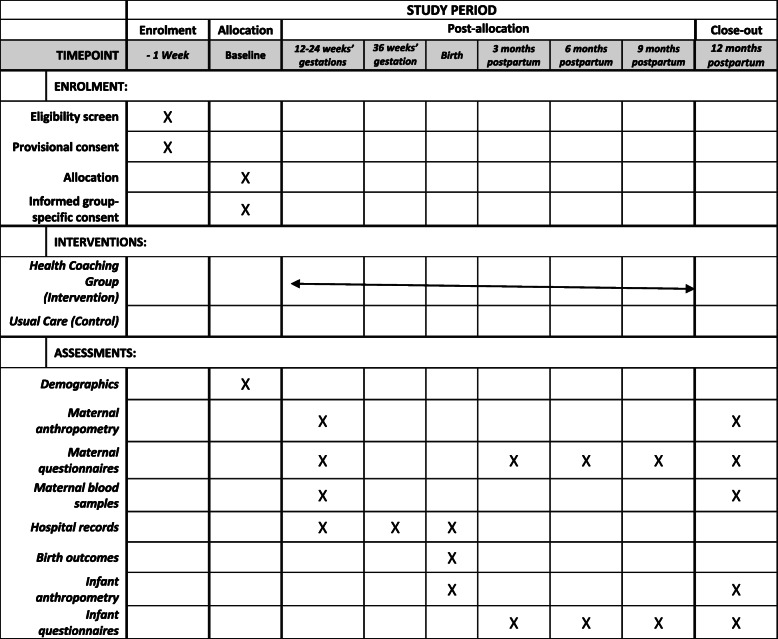
Standard Protocol Items: Recommendations for Interventional Trials (SPIRIT) timeline providing information about participant enrolment, intervention, and outcomes evaluated during the trial according to SPIRIT recommendations

This protocol is reported in accordance with the Standard Protocol Items: Recommended items to address in a clinical trial protocol and related documents (SPIRIT) guidelines. The SPIRIT checklist is available in Additional file 1, and the timeline figure is detailed in Fig. 2.

### Population

Pregnant women aged 18 and older attending maternity services within any of the hospital sites will be screened for their risk of developing GDM using the validated Monash GDM Screening Tool [28] upon referral to the hospital by the research staff. Individuals scoring at risk (3 or higher) will be further assessed as to whether they meet the additional study criteria.

The following are the inclusion criteria:

- Women attending one of the five participating maternity hospitals for their maternity care
- Women identified at high risk of developing GDM: defined by the Monash GDM Screening Tool score of 3 or higher [28]
- Women less than 24 weeks’ gestation
- Women owning a smartphone capable of hosting the intervention app
- Women not currently participating in any other lifestyle-related clinical trial

The following are the exclusion criteria:

- Established diabetes (T1DM or T2DM), previously known
- Current multiple pregnancy (e.g. twin, triplets)
- Cancer (not in remission)
- Severe mental illness in the last 3 months
- Substance abuse (illicit drugs) in the last 3 months
- Myocardial infarction in the last 3 months
- Inability to verbally communicate in English for the Irish, English, and Australian sites and inability to verbally communicate in Spanish for the Spanish site

If a woman is deemed eligible for study inclusion by the research team, they will receive a preliminary participant information sheet about the study and have the opportunity to discuss with a member of the research team before providing written informed consent and enrolling in the study. As the trial and intervention are embedded in usual antenatal care, some site-specific adaptations were made which are highlighted in the figure and methods where relevant.

### Randomisation procedure

Participants will be randomised to the intervention or control group at enrolment to the study in advance of their first antenatal visit. The randomisation will be generated centrally by the trial biostatistician using a computer-generated random sequence on a 1:1 basis using block randomisation of 200 participants for each site. Allocation will be recorded in sequentially numbered and sealed, opaque envelopes, distributed to study sites. For each participant, the researcher at each site will select and open the next sequential envelope, with an independent witness and co-sign the envelope. As the Melbourne site delivers their first antenatal appointment online, the randomisation sequence will be embedded into the study management database and each participant will be assigned sequentially as they are registered to the study. The envelopes for this site will be retained as a randomisation quality check. At the first study visit, participants will receive an arm-specific information sheet and have further opportunities to ask any questions. Informed written consent to participate will then be secured. Throughout the study, individual participants will only be provided with the information relevant to their arm of the study and will not be made overtly aware of their allocation to control or intervention groups. Study clinicians who are providing services to the participants and researchers carrying out data collection will not be informed by the research team as to their allocation to control or intervention groups.

### Sample size

The study is an effectiveness trial and will use intention-to-treat analysis. Based on the primary outcome of maternal weight status at 12 months postpartum, to detect a 0.8-kg/m^2^ BMI difference between the groups with a standard deviation of 2 kg/m^2^ BMI, a type I error rate of 5%, and to achieve 80% power, 800 women need to be recruited to the trial (i.e. 400 in each arm of the study). This incorporates an assumed retention rate of 70% which is more conservative than previous studies to allow for country-level variance in retention. Each site will recruit 200 women over a 12-month period. Based on previous experience recruiting women from this setting and examining the potential numbers of eligible women using the current Monash Health rate of women scoring 3 or higher on the GDM screening tool (estimated to be approximately 33% prevalence), it is anticipated that recruitment will take around 12 months.

The study flow is described in Fig. 1.

### Intervention

**Fig. 3:**
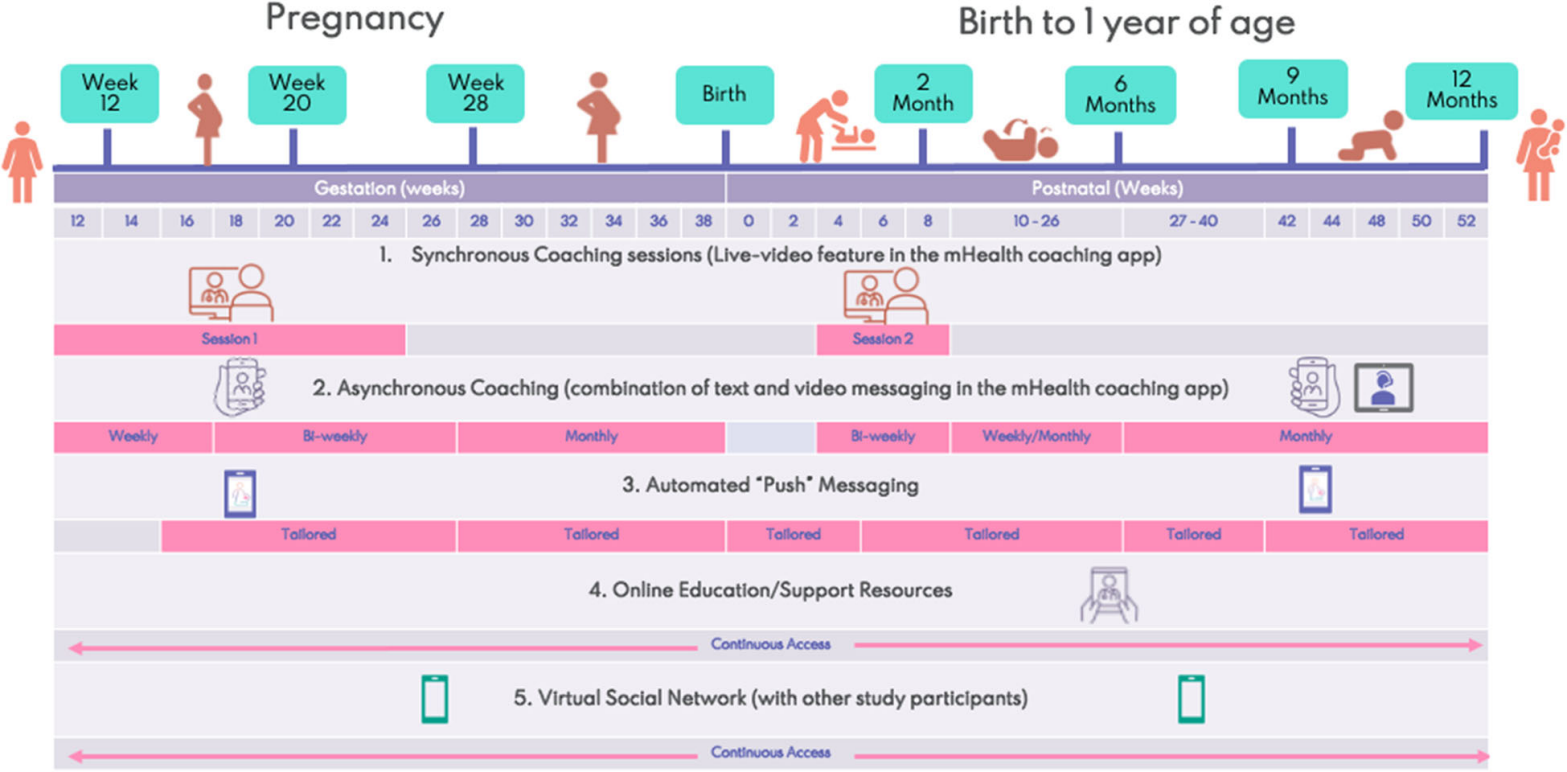
Summary of the Bump2Baby and Me intervention components and frequency of each in the app

The intervention group will receive mHealth coaching support from enrolment in the study until 1 year postpartum. The mHealth coaching will be delivered by trained health coaches with a healthcare professional background including nutrition, nursing, physiotherapy, or health psychology. The coaches will drive the woman’s engagement with the app content and personalise their behaviour change experience based on their own goals and needs. The smartphone app content and the health coach training programme are based on previous research from RCTs which were effective at reducing unhealthy weight gain in both the mother and child [16, 17, 27]. The intervention incorporates content from the PEARs and MADGA RCTs which were effective in reducing maternal weight gain in pregnancy and postnatally respectively and My Baby Now study which is based on the InFANT program RCT, effective in influencing child obesity risk behaviours [27, 29]. Each intervention was designed using behaviour change theory; the Behaviour Change Wheel theoretical framework was overlaid onto the app development to ensure complete and cohesive coverage of techniques [30]. The app and health coach environment are supported by a developed and tested commercial platform that uses a health coach dashboard to coordinate and tailor engagement at the individual level. The platform will integrate seamlessly with a woman’s smartphone, and two local health coaches will deliver the intervention at each site. A summary of the components and frequency is detailed in Fig. 3.

The intervention consists of six components as follows:

1. Synchronous mHealth coaching: Coaching sessions are conducted on a 1:1 basis between the mHealth coach and participants. There will be 2 synchronous sessions, the first at enrolment and the second between 4 and 8 weeks postpartum. These sessions will last 45–50 min with a follow-up summary video message of the goals discussed and, at the first session, the establishment of a behaviour change agreement. These sessions are mediated through a live-video feature in the mHealth coaching app. If the woman is diagnosed with GDM, there will be an opportunity for a third 15-min synchronous coaching session to review and adjust any lifestyle goals to align with the individual’s diabetes in the pregnancy management plan.
2. Asynchronous mHealth coaching: This uses a combination of text and video messaging exchanges between the mHealth coach and participant and tailored to the woman’s needs. The pregnancy interactions will be once a week for the first 4 weeks. The mHealth coaching then becomes bi-weekly for 2 months and then monthly until birth. The postpartum interactions will be after the second synchronous coaching session. These will happen bi-weekly for 1 month, to support the woman to ease back into mHealth coaching contact after the birth. After that, it will be weekly for a month, then bi-weekly for a month prior to monthly check-ins with the mHealth coach. However, this will be arranged with the woman during the postpartum synchronous coaching session and will be adapted accordingly. From 6 to 12 months postpartum, asynchronous coaching will happen monthly.
3. Automated push notifications: These will include messages prompting the individual to follow through on the goals they have set for themselves, reminders to register goal achievements, and motivational messages when they have accomplished their goals. Whilst these are standardised messages, their frequency of delivery will be tailored to match the individual’s goals. For example, if a woman sets a weekly goal to exercise, they will receive a weekly push reminder about this goal.
4. Personalised educational content: Provided by the mHealth coach during the asynchronous coaching sessions, this content will cover topics within navigating the healthcare system and antenatal appointments, healthy eating for pregnancy and postpartum, physical activity, emotional well-being, breastfeeding, and best practice formula feeding. Each mHealth coach will continuously assess what content is relevant before sending it to the women.
5. Additional support content: Participants will also receive automated push notifications referring to additional content available in the app. The content push notifications will be active once a week during the weeks that no asynchronous mHealth coaching occurs. These push notifications will connect participants with specially designed online resources providing tailored information such as recipes, tips for food and activity choices, breastfeeding resources, and links to relevant support agencies.
6. Virtual social network: Participants will have access through the mHealth coaching app to a network with other women participating in the intervention arm of the study where messages can be sent. This will enable social engagement and support, as well as the capacity to connect physically for shared activities, self-organised in each study site.

### Control

The control group participants will be provided with standard written information for their site and links to approved country-specific national websites on the prevention of gestational diabetes, managing gestational weight gain, encouraging breastfeeding, postpartum weight loss, and diabetes prevention. They will also be provided with the details of the Bump2Baby website (www.bump2babyandme.org) which contains information in line with standard care for each site.

To improve retention throughout the study, both groups will receive electronic newsletters updating them on the progress of the study, and reminders to access the Bump2Baby website information, as they progress through their pregnancy and first year of motherhood. All study information will also be available on the website.

### Outcome measurements

The primary outcome is a reduction of 0.8 kg/m^2^ maternal BMI in the intervention group at 12 months postpartum.

The secondary outcomes will include the differences in the following measured during pregnancy and postpartum (Table 1):

- Gestational weight gain and weight status
- Maternal blood pressure
- Maternal physical activity and sleep
- Maternal psychological health
- Maternal and infant diet
- Metabolic markers including blood glucose and blood lipids
- Glycaemic status and GDM diagnosis
- Birth data including mode of delivery, birth weight, placental weight, and any complications
- Newborn and infant anthropometry (weight centiles, BMI *z*-scores)
- Breastfeeding (any and exclusivity) and duration
- Infant development
- Infant physical activity and sedentary time

**Table. 1:** Domains and assessment questionnaires for secondary analyses in the Bump2Baby and Me Study

Domain	Measurement/questionnaire	Details	Time point measured	References
**Maternal**				
**Diet**	Food4Me Food Frequency Questionnaire (FFQ)	Contains questions on the average consumption of 157 food items over the previous month. Food portion size is estimated using photographs. The questionnaire has been validated against standard food frequency questionnaires and 4-day weighed food diaries, in multiple countries including Ireland, Spain, and the UK. **Measurements units**: Continuous: nutrient values Categorical: meeting dietary guidelines **Analysis:** Compare mean/median and SD/IQR and meeting guidelines (yes/no)	- Pregnancy (12–24 weeks’ gestation) - Postpartum (12 months)^a^	[31]
** Physical activity**	1. Pregnancy Physical Activity Questionnaire (PPQA) 2. Smartphone app inbuilt activity tracker (intervention group only)	1. The PPQA was identified as the most psychometrically sound self-reported measure of physical activity in pregnancy. It is available in English and Spanish. 2. The smartphone inbuilt activity tracking capabilities of individuals phones will be used to record physical activity for the intervention group. This will be used to record the estimated step count for participants. **Measurement units:** Continuous: metabolic equivalent minutes (METs), Time spent in moderate/vigorous physical activity, steps per day. Categorical: Meeting physical activity recommendations **Analysis:** Compare mean/median and SD/IQR and meeting guidelines (yes/no)	- Pregnancy (12–24 weeks’ gestation) - Postpartum (12 months)^a^	[32, 33]
**Breastfeeding**	1. Iowa Infant Feeding Attitudes Scale (IIFAS) 2. Adapted version of the Australian National Infant Feeding Survey 2010 and InFANT Extend Study	1. The IIFAS has been validated in English and Spanish, with good reliability. 2. Adapted questions will be used from the Australian National Infant Feeding Survey and InFANT Extend Study to assess levels of breastfeeding, feeding styles (including combinations of breast milk and formula), experience of pumping, timing and frequency of feeding, and age of weaning. **Measurement units:** Continuous: IIFA score (17–85) Categorical: IIFAS scores will be categorised into groups: (1) positive to breastfeeding, (2) neutral, and (3) positive to formula feeding. Groups will be created based on previous breastfeeding experience, breastfeeding intentions, and meeting breastfeeding and infant feeding recommendations. **Analysis:** Compare the mean/median and SD/IQR and categories	- Pregnancy (12–24 weeks’ gestation) - Postpartum (3, 6, 9, 12^a^ months)	[34–37]
**Health status**	EuroQol-5 Dimension (EQ 5-D-L)	The EQ 5-D-L is a standardised instrument for measuring generic health status and has been extensively validated in over 200 countries. **Measurement units:** Continuous: self-rated EQ visual analogue scale (1–100), score 1–5 for five dimensions: mobility, self-care, usual activities, pain/discomfort, and anxiety/depression (score 1–5) Categorical: high/low across the five dimensions **Analysis:** Compare scores in categories, mean/median, and SD/IQR for visual analogue scale	- Pregnancy (12–24 weeks’ gestation) - Postpartum (12 months)^a^	[38, 39]
**Psychological health**	Edinburgh Postnatal Depression Scale (EPDS)	The EDPS is a questionnaire originally developed to assist in identifying possible symptoms of depression in the postpartum period. It also has adequate sensitivity and specificity to identify depressive symptoms in the antenatal period and is useful in identifying symptoms of anxiety. The EDPS has been extensively validated in multiple languages including Spanish, with good sensitivity, reliability and established validity against diagnostic interviews. **Measurement units:** Continuous: score (1–25) Categories: at-risk of depression (< 13, yes/no) **Analysis:** Compare the mean/median and SD/IQR and categories	- Pregnancy (12–24 weeks’ gestation) - Postpartum (3, 6, 9, 12^a^ months)	[40–42]
**Sleep quality**	Pittsburgh Sleep Quality Index (PSQI)	The PSQI is a self-report questionnaire that assesses sleep quality over a 1-month time interval. The PSQI is one of the most widely used sleep questionnaires and has been translated and validated into multiple languages, including Spanish, and amongst antenatal and postpartum populations. **Measurement units:** Continuous: total score (0–21), 7 subscores (0–3) Categories: poor sleep quality (> 5, yes/no) **Analysis:** Compare the mean/median and SD/IQR and categories	- Pregnancy (12–24 weeks’ gestation) - Postpartum (3, 6, 9, 12^a^ months)	[43–45]
**Health literacy**	Health Literacy Questionnaire (HLQ) and electronic Health Literacy Questionnaire (eHLQ)	The HLQ and eHLQ are a series of independent scales that assess functional health literacy and the fuller constructs of health literacy. The HLQ has extensive cross-cultural validity and has translations available in Spanish. The eHLQ is well-defined, validated, and reliable and was designed to evaluate interactions with digital health services. **Measurement units:** Continuous: 9 subscales scores (1–5) Categories: adequate/inadequate health literacy **Analysis:** Compare the mean/median and SD/IQR and categories	- Pregnancy (12–24 weeks’ gestation) - Postpartum (12 months)^a^	[46, 47]
**Willpower**	6 Implicit Theories of Willpower – Resisting Temptation (ITW-RT)	The ITW-RT scale has been shown to have good psychometric properties, including predictive and concurrent validity. **Measurement units:** Continuous: total score (1–30) Categories: non-limited theory of willpower (score 0–17), limited theory of willpower (18–30) **Analysis:** Compare the mean/median and SD/IQR and categories	- Pregnancy (12–24 weeks’ gestation) - Postpartum (12 months)^a^	[48–50]
**Infant**				
**Development**	Ages & Stages Questionnaires, Third Edition (ASQ-3)	The ASQ-3 is a parental-reported developmental screening tool that shows high reliability, internal consistency, sensitivity, and specificity. It is available in English and Spanish. **Measurement units:** Continuous: 5 subscale scores (5 areas; communication, gross motor, fine motor, problem solving, personal-social) Categories: typical development or need for monitoring/further assessment **Analysis:** Compare the mean/median and SD/IQR scores of sub scales and categories	- Postpartum (12 months)	[51, 52]
**Physical activity**	Two question items—sedentary/screen time and outdoor play	Infant physical activity at 12 months of age will be assessed using two question items: one on sedentary time including screen time over a typical week and time spent in situations that restrict movement (e.g. stroller, playpen) and one on the amount of activity about the number of hours the child typically spends playing outdoors on weekdays and weekend days. **Measurement units:** Continuous: time spent sedentary or playing (minutes) Categories: meeting guidelines **Analysis:** Compare the mean/median and SD/IQR and meeting guidelines (yes/no)	- Postpartum (12 months)	
**Diet**	Complementary Food Frequency Questionnaire (CFFQ)	The CFFQ comprises 49 food items under six food categories which measures what the infant consumed over the last 4 days. Breastmilk and formula intakes are also measured in this validated tool. **Measurement units:** Continuous: nutrient values Categories: meeting dietary guidelines **Analysis:** Compare the mean/median and SD/IQR and meeting guidelines (yes/no)	- Postpartum (12 months)	[53]
**Health status**	Patient-Oriented SCORing Atopic Dermatitis (PO-SCORAD)	Incidence of infections, eczema, and atopic dermatitis will be assessed using the PO-SCORAD, a self-assessment tool for use by parents which has been validated in 12 European countries. **Measurement units:** Continuous: PO-SCORAD Score (0–90) Categories: incidence of infections, eczema, and atopic dermatitis (yes/no) **Analysis:** Compare the mean/median and SD/IQR and incidences	- Postpartum (12 months)	[54]
**Health costs**	Adapted questionnaires from the InFANT Extend Study and the Infant Feeding Practices Study II	Participant employment, childcare, and out of pocket costs will be estimated by self-completed questionnaires derived from the Infant Feeding Practices Study II. Health costs for the child will be assessed using an adaption of the instrument used to assess costs in the InFANT Extend Study. The questionnaire covers health costs, travel costs, and lost productivity during the antenatal and postpartum periods. **Measurement units:** Continuous: productivity loss (absenteeism and presenteeism), out-of-pocket costs Categorical: employment status, childcare status, high/average/low costs, and productivity losses **Analysis:** Compare the mean/median and SD/IQR and groups	- Postpartum (3, 6, 9, 12^a^ months)	[37, 55]
**Sleep**	Brief Infant Sleep Questionnaire (BISQ)	Sleep duration, night wakings, and method of falling asleep will be measured using the BISQ which is available in English and Spanish. **Measurement units:** Continuous: time spent asleep/awake (minutes) and sleep behaviour scores Categorical: problem sleeper (yes/no), meeting sleep recommendations (yes/no) **Analysis:** Compare the mean/median and SD/IQR and groups	- Postpartum (3, 6, 9, 12^a^ months)	[56]

For measurements collected at more than one time point

^a^The primary outcome of interest

### Baseline assessments

Participants will attend the first study visit after recruitment in line with routine antenatal care. A researcher will facilitate participants in the intervention group with onboarding, including downloading the mHealth coaching app, account creation, and scheduling the initial coaching session. For participants in the control group, the research clinician will provide the women with a written leaflet with details of the approved website and show the woman how to access the health information on the Bump2Baby website. Demographic characteristics will be collected via questionnaire and will include maternal age, ethnicity, gravidity, parity, relationship status, educational attainment, employment status (of the participant and relevant partner), housing status, childcare responsibilities, and prior medical history.

Anthropometric measurements of maternal height (cm) and weight (kg) will be extracted from medical records, and all participants will also be provided with a set of digital scales (Smart Body Fat Scale, RENPHO©) for the duration of the study. Participants will be provided with a manual on how to use the scales appropriately and will be asked to weigh themselves approximately once a week. The scales will connect via Bluetooth, with an app on the participant’s phone, which facilitates automatic recording of this data when the individual is connected to a wireless network. Body mass index (BMI, kg/m^2^) will be calculated from these measurements. Blood pressure will also be measured using a calibrated blood pressure monitor at the baseline visit.

Participants will be asked to complete the online questionnaires, detailed in Table 1, within 1 week of the baseline visit before the first health coaching session for the intervention group.

### Antenatal assessments

Weight will continue to be recorded weekly by participants using the digital scales provided, and gestational weight at 36 weeks (or closest time point) will also be obtained from medical records. If the participant has had an Oral Glucose Tolerance Test (OGTT) at 24–26 weeks’ gestation within their usual care, this result will be taken from the medical records. Foetal ultrasound scan results will also be obtained from medical records, where available. The measurements recorded, where available, will include crown rump length (mm), gestational age (weeks/days at scan), estimated due date, biparietal diameter (mm), abdominal circumference (mm), femur length (mm), estimated foetal weight (g), liquor volume, amniotic fluid index (max pool depth, cm), abdominal fat mass (mm), subscapular fat mass (mm), mid-thigh lean mass (cm^2^), mid-thigh fat mass, umbilical artery pulsatility index, umbilical artery resistive index, and end-diastolic flow.

### Birth assessments

After birth, the following details will be obtained where available from the medical records: gestation (weeks), maternal weight (closest collected to delivery from medical records, kg), delivery method, (including any medical assisted interventions), birth complications (including admission to the special care nursery and/or neonatal hypoglycaemia), placenta weight (g), Apgar score, and anthropometry (birth weight (g), length (cm), and head circumference (cm)).

### Postpartum assessments

It will be encouraged that weight will continue to be recorded weekly by participants using the digital scales provided. Breastfeeding will be assessed using an adapted version of the Australian National Infant Feeding Survey 2010 and InFANT Extend Study questionnaires to assess the history of breastfeeding, and for those that breastfed, their experience of feeding, pumping, and timing over the 3 monthly intervals (3, 6, 9, 12 months) [36, 37]. Psychological health will be assessed by the Edinburgh Postnatal Depression Scale (EPDS) and sleep quality by the Pittsburgh Sleep Quality Index (PSQI) at the same intervals [38, 43]. Parental-report infant weight (kg), length (cm), and head circumference (cm) will be collected at 3, 6, and 9 months. All metrics and questionnaires being completed postpartum are detailed in Table 1.

### 12-month postpartum assessment

At 12 months postpartum, participants will be contacted by the research team to arrange the last study visit. Participants will attend with their infant, and the following measurements will be collected: infant length (cm, assessed using a calibrated length mat), weight (kg), and head circumference (cm). Maternal height will be measured in centimetres and weight in kilograms. Standard operating procedures will be provided for these measurements. BMI *z*-score will be calculated using WHO growth standards [57].

Participants will be asked to complete further questionnaires, as detailed in Table 1, online the week before this appointment.

### Biological samples

Non-fasting blood samples will be taken by phlebotomists or research nurses at the baseline visit (first antenatal appointment) and at the 12-month postpartum visit. Where routine OGTTs are performed antenatally (24–26 weeks) or postpartum (6–12 weeks), an additional blood sample will be taken at the fasting time point for analysis. These will be processed and stored at − 80 °C at the local site until secure transport of all samples to the University College Dublin for assay using standard procedures and laboratory equipment. All assaying will be performed at study completion and will include the following metabolic markers: HbA1c, glucose, and blood lipids (total cholesterol, HDL cholesterol, LDL cholesterol, triglycerides).

### Additional intervention assessments

The following assessments will be carried out in the intervention arm of the RCT:

- Fidelity assessment delivery: An mHealth coaching manual has been prepared to detail the a priori behaviour change techniques to be used by the coaches in the synchronous and asynchronous coaching interactions at different determined time points. All coaching interactions, synchronous and asynchronous, will be recorded, in either video or text format as per the type of interaction. A sample of these interactions will be coded and compared to the coaching manual, on an ongoing basis, to provide a continuing feedback fidelity process to the mHealth coaches. For a random sample of participants, all coaching interactions will be coded against the specified coaching manual to generate a fidelity index, so that treatment fidelity can be examined in relation to participant outcomes.
- Exposure to intervention: All automated and non-automated motivational push notification messages and reminders from the mHealth coaching app and coach will be recorded. The mHealth coaching app platform records which messages within the app are accessed by participants, and this data will be used to determine the total number of messages received by everyone, by message content (information, motivational, reminder). This will be used to generate an exposure index for each message type, for which the denominator will be the highest number of messages received by any one individual participant.
- Experience of intervention: Qualitative analysis will be carried out to assess participant experience of the study. A subset of participants and a support person (mother, mother-in-law, sibling, friend, or partner as determined by the woman) will be invited to participate in an interview upon completion of the 12-month postpartum follow-up visit. Approximately twenty participants per country are estimated to be required to achieve thematic congruence. This short interview (15–30 min) will explore their study experience, which will include their perception of the study, their experience of the impact, perceptions of core elements of the programme that were central to their experience, and any recommendations for further development of the intervention.

The RCT sits within the larger implementation project, and as such, much greater detail has been collected on the implementation aspects prior to the RCT commencing and the broader setting. Briefly, the project used the EPIS framework to scaffold the work [58]. The framework consists of 4 main components: exploration, preparation, implementation, and sustainment. The exploration phase involved evaluation of current needs to ensure the intervention fit across system levels, organisation levels, and staff and recipient levels. The preparation phase involved planning for the intervention through training, service mapping, and awareness. The implementation will be assessed using the RE-AIM framework [59], which measures results along the dimensions of Reach (to specified patient groups), Effectiveness (of the Bump2Baby and Me RCT), Adoption (by practice settings and clinicians), Implementation fidelity (consistency of delivery by various staff), and Maintenance (i.e. practices and results over the long term). Longitudinal interviews of key stakeholders at each clinical site will be carried out at quarterly intervals during the project. These semi-structured interviews will assess the local context and environment to measure what is happening within the hospital clinics and services that might impact the intervention and how well it fits with the current routine care. Qualitative thematic analysis of these interviews will enable the exploration of the Implementation and Sustainment components of the EPIS framework. This will be reported in an additional more detailed implementation protocol for the overall study.

### Statistical analyses

The intervention arm will be tested against usual care. Intention-to-treat (ITT) and a per-protocol set (PPS) analysis will be planned for both primary and secondary outcomes. Full analysis set is defined according to the ITT principle and will consist of all randomised subjects analysed according to the study arm to which they were assigned at randomisation, except for subjects who were randomised to the study in error and were subsequently excluded, e.g. twin pregnancies. The per-protocol set (PPS) will consist of all patients in the full analysis set without a major protocol deviation. Major protocol deviations include, but are not limited to, the following:

- The subject was assigned to the intervention arm but did not reach minimum exposure. The minimal exposure requirement is that participants received the first onboarding session with the health coach.
- The subject did not have a post-baseline assessment.
- An eligibility criterion that was deemed to have been met at the time of randomisation was later found not to have been met.

Baseline characteristics will be compared between the intervention and control groups using the *χ*^2^ tests, the independent *t*-tests, and/or the Wilcoxon rank-sum test, as appropriate. Primary and secondary outcomes will be evaluated using repeated-measures analysis of variance (ANOVA) or mixed model analyses. Two-sided tests will be used, with a level of *P* < 0.05 determining statistical significance. Comparisons of the assessment times (the baseline and 12 months postpartum differences) within each treatment group (control or intervention) will be based on *t*-tests that utilise the standard errors of the differences that are computed as part of the repeated-measures analysis. Covariates included in the model will be kept to a minimum—site is the only covariate expected. The impact of loss to follow-up will be minimised by using a marginal model, which is not subject to list-wise deletion: each participant will contribute all time-specific measurements made to the model. Missing data will be reported for each outcome and handled by applying multiple imputation methods [60]. Subgroup analyses will be carried out to investigate the site differences based on the substantial demographic and operative differences in the four clinical sites. There are no interim analyses planned. Analyses will be conducted using the IBM SPSS software (version 26.0 or later; IBM), STATA version 12 or later (Stata Corp., College Station, TX, USA), or SAS software (SAS Institute Inc., Cary, NC, USA), as appropriate for analysis type. Data analysts will be blinded to group assignments.

A statistical analysis plan and data management plan have been prepared for the project which outlines plans for data entry, coding, confidentiality, security, and analysis in line with best practice (Additional file 2). Each clinical site is incorporated in this data management plan and provided the details to their relevant ethics committee. The project has an independent data manager who oversees the data monitoring and analyses and reports back to the RCT Working Group. There is no formal data monitoring committee; however, the external advisory board receives reports from the data manager.

## Discussion

The multicentre Bump2Baby and Me RCT will assess the impact of a low-resource, evidence-based mHealth intervention in the form of a smartphone application, which extends across both the pregnancy and postnatal period, in improving health outcomes for mothers and infants. The project will identify women at high risk of developing GDM using a validated screening tool and support them using personalised health coaching via a smartphone application which builds on previous research that has been shown to successfully reduce inappropriate weight gain in three separate RCTs [16, 17, 27].

The Bump2Baby and Me RCT protocol has been designed to fit within the existing antenatal care settings, and local adaptations were made to ensure that the trial could proceed alongside changes that occurred to maternity services due to the severe acute respiratory syndrome coronavirus 2 (SARS-CoV-2) outbreak. Recruitment of participants will occur remotely at all clinical sites, apart from Granada, to reduce face-to-face contact, and the first study visit is embedded within the routine antenatal visit to avoid further burden on the participants. With the health coaching intervention occurring virtually through a mobile phone application, this trial is particularly well-suited in providing support to pregnant women in an accessible and safe way. This project is particularly timely with the move towards an increasingly digital health service with the intervention being appropriate and suitable for use in providing additional support during antenatal and postnatal care post-pandemic and in future health systems.

This intervention employs precision medicine supported by behaviour change and personalised coaching with a concurrent mixed-methods implementation research approach. Previous interventions have not moved beyond the tightly controlled research trial phase to consider implementation into routine service delivery, and this multicentre project aims to provide a roadmap for this that is universally acceptable. It is anticipated that this study will contribute to the early prevention of maternal and child diabetes, overweight, obesity, and other non-communicable diseases on a global scale.

## Trial status

This publication is based on protocol version 1, 14 January 2021. Recruitment for the trial began on 9 February 2021, and recruitment at all sites is anticipated to be completed by February 2022.

## Supplementary Information

**Additional file 1.** SPIRIT checklist.

**Additional file 2.** Bump2Baby and Me.

## Data Availability

The trial dataset, data management plan, protocol, and study materials will not be made publicly available. Researchers who wish to access full study materials and/or dataset may be granted access upon reasonable request to the corresponding author. We do not intend to use professional writers. The *International Committee of Medical Journal Editors* criteria will be used to determine authorship eligibility: • Substantial contributions to the conception or design of the work or the acquisition, analysis, or interpretation of the data for the work • Drafting the work or revising it critically for important intellectual content • Final approval of the version to be published • Agreement to be accountable for all aspects of the work in ensuring that questions related to the accuracy or integrity of any part of the work are appropriately investigated and resolved A detailed dissemination policy has been prepared for the project. This includes publication guidelines which set out the authorship criteria for intended publications. Findings will be published in peer-reviewed journals and disseminated through various channels including social media and at national and international meetings. Study findings will also be prepared in lay terms for study participants and the general public and will be disseminated through the study newsletters and on the project website (https://bump2babyandme.org/).

## Abbreviations

ANOVA: Analysis of variance
BMI: Body mass index
GDM: Gestational diabetes mellitus
ITT: Intention to treat
OGTT: Oral Glucose Tolerance Test
PPS: Per-protocol set
RCT: Randomised controlled trial
T2DM: Type 2 diabetes mellitus
WHO: World Health Organization
