# Supplementary material for: Bump2Baby and Me: protocol for a randomised trial of mHealth coaching for healthy gestational weight gain and improved postnatal outcomes in high-risk women and their children
Source: Trials. 2021 Dec 28;22:963. doi: 10.1186/s13063-021-05892-4 (PMC8713543; doi:10.1186/s13063-021-05892-4)
Supplement: Supplementary file 2 — Additional file 2. Bump2Baby and Me. [file 13063_2021_5892_MOESM2_ESM.pdf]

Ref. Ares(2020)2300133 - 30/04/2020

Bump2Baby and Me

Implementation action to prevent diabetes from Bump 2 Baby (IMPACT DIABETES B2B): A low-resource system of care intervention for appropriate gestational weight gain and improved postnatal outcomes

Work Program: Horizon 2020

Call: H2020-SC1-BHC-2018-2020

Grant Agreement Number: 847984

Start Date: 01/01/2020

Duration: 60

Project Website: www.bump2babyandme.org

Deliverable Report	
Deliverable	D7.3
Deliverable Name	Data management plan
Deliverable Status	Final
Responsible Partner [No.]	NUID UCD [1]
Due date of deliverable	30/04/2020
Actual deliverable date	30/04/2020
Justification if delayed	N/A

Dissemination Level of this Report		
PU	Public	X
PP	Restricted to other programme participants (including the Commission Services)	
RE	Restricted to a group specified by the consortium (including the Commission Services)	
CO	Confidential, only for members of the consortium (including the Commission Services)	

This project has received funding from the European Union's Horizon 2020 research and innovation programme under grant agreement No 874984.

IMPACT DIABETES B2B Consortium is composed of:		
NUID UCD[1]	University College Dublin	Ireland
DEAKIN	Deakin University	Australia
UCPH	University of Copenhagen	Denmark
UNIVBRIS	University of Bristol	UK
MONASH	Monash University	Australia
LIVA	Liva Healthcare	Denmark
UGR	University of Granada	Spain
BETA	Beta Technology	UK
AU	Aarhus University	Denmark
NBNHS	North Bristol Nation Health Service Trust	UK

[1] Project Coordinator

Disclaimer

This document reflects only the author's views and the European Union is not liable for any use that may be made of the information contained therein.

Authors List

Lead Author (Editor)			
Surname	First Name	Beneficiary	Contact Email
O'Reilly	Sharleen	NUID UCD	sharleen.oreilly@ucd.ie
Co-Authors (in alphabetical order)			
Surname	First Name	Beneficiary	Contact Email
Skinner	Timothy	UCPH	ts@psy.ku.dk
Salter	Mitchell	BETA	mitchell.salter@betatechnology.co.uk

Revision Control

Version	Author	Date	Status
1.0	NUID UCD	10/03/20	Initial draft
1.1	NUID UCD	17/04/20	Draft sent to partners for feedback
2.0	NUID UCD	30/04/20	Final draft reviewed
2.1	NUID UCD	30/04/20	Submission to the EC

Contents

List of Tables 6
List of Definitions and Abbreviations 6
Executive Summary 7
1. Introduction 8
2. Data summary 9
2.1 Purpose of data collection/generation and relation to project objectives 9
2.2 Types and formats of data generated/collected 10
2.3 Specify if existing data is being re-used (if any) 11
2.4 Specify the origin of the data 11
2.5 State the expected size of the data 11
2.6 Outline the data utility: to whom will it be useful 11
3. FAIR data 12
3.1 Making data findable, including provisions for metadata 12
3.1.1 Outline the discoverability of data (metadata provision) 12
3.1.2 Outline the identifiability of data and refer to standard identification mechanism 12
3.1.3 Outline naming conventions used 12
3.1.4 Outline the approach towards search keyword 13
3.1.5 Outline the approach for clear versioning 13
3.1.6 Specify standards for metadata creation 13
3.2 Making data openly accessible 13
3.2.1 Specify which data will be made openly available 13
3.2.2 Specify how the data will be made available 15
3.2.3 Specify methods or software tools are needed to access the data 15
3.2.4 Specify where the data and associated metadata, documentation and code are deposited 15
3.2.5 Specify how access will be provided in case there are any restrictions 15
3.3 Making data interoperable 15
3.4 Increase data re-use 16
3.4.1 Specify how the data will be licenced to permit the widest reuse possible 16
3.4.2 Specify when the data will be made available for re-use 16
3.4.3 Specify whether data produced and/or used in the project is useable by third parties 16
3.4.5 Describe data quality assurance processes 17
3.4.6 Specify the length of time for which the data will remain re-usable 17
4. Allocation of resources 18
4.1 Estimated costs for making data FAIR 18
4.2 Clearly identify responsibilities for data management 18
4.3 Describe costs and potential value of long term preservation 18
5. Data security 19
5.1 Data confidentially and integrity 19

5.2 Data availability 19
6. Ethical aspects 20
7. Other 21
Annex 1 Data inventory tables 22
Summary 22
Metadata provision 22
Storage of data 22
Re-use of data 22

List of Tables

Table 3.1 Expected levels of accessibility for IDB2B data

List of Definitions and Abbreviations

Abbreviation	Definition
CA	Consortium Agreement
D	Deliverable
DMP	Data Management Plan
EC	European Commission
EU	European Union
GA	Grant Agreement
GDM	Gestational Diabetes Mellitus
H2020	Horizon 2020
IDB2B	IMPACT DIABETES B2B
SC	Steering Committee
WP	Work Package

Executive Summary

The present document is a deliverable of the IMPACT DIABETES B2B (IDB2B) project, funded by the European Commission (EC) Directorate-General for Research and Innovation under its Horizon 2020 Research and Innovation programme (H2020).

This Data Management Plan (DMP) defines how data associated with the IDB2B project will be identified, analysed, and managed. It outlines how data management activities will be performed, recorded, and monitored throughout the lifetime of the project.

This deliverable will be progressed within other deliverables within this Work Package (WP). The Annex provides templates for data inventory tables.

1. Introduction

Open Science and Innovation is critical to ensuring science is more responsive and efficient to the challenges presented by a constantly changing environment and knowledge base. Open Science does not mean that all data is freely available all of the time. It is essential to ensure that intellectual property is protected before making knowledge openly available to the public as it may need to be used within innovation.

The European Union enables Open Innovation by requiring that projects funded under the European Union Framework Programme for Research and Innovation comply with making data as open as possible. Open access can be defined as the practice of providing on-line access to scientific information that is free of charge to the reader. In the context of R&D, open access typically focuses on access to '*scientific information*' or '*research results*', which refers to two main categories:

- Peer-reviewed scientific research articles (primarily published in academic journals)
- Research data

Horizon 2020 (Article 29) mandates open access to all scientific publications and, from January 2017, to all research data, with the possibility to opt out from this “Open Research Data” pilot. An electronic copy of the publication must be deposited in a suitable green open access repository.

Under Horizon 2020, each beneficiary must ensure open access to all peer-reviewed scientific publications relating to its results.

Beneficiaries can freely choose between the most appropriate route towards open access for them:

- Self-archiving (also referred to as 'green' open access) means that a published article or the final peer-reviewed manuscript is archived (deposited) in an online repository before, alongside or after its publication. Repository software usually allows authors to delay access to the article ('embargo period') If this route is chosen beneficiaries must ensure open access to the publication within a maximum of six months (twelve months for publications in the social sciences and humanities).
- Open access publishing (also referred to as 'gold' open access) means that an article is immediately provided in open access mode (on the publisher/journal website). Publishers sometimes charge so called Article Processing Charges (or APCs) to make articles open. Such costs are eligible for reimbursement during the duration of the project as part of the overall project budget. In the case of gold open access publishing, open access must be granted *at the latest on the date of publication* and you also have to deposit a copy in a repository.

In Horizon 2020 the Commission launched a flexible pilot for open access to research data (ORD pilot). The pilot aims to improve and maximise access to and re-use of research data generated by Horizon 2020 projects, taking into account

- the need to balance openness and protection of scientific information
- commercialisation and IPR
- privacy concerns
- security
- data management and preservation questions

As of the Work Programme 2017 the Open Research Data pilot is extended to cover all thematic areas of Horizon 2020 per default. However, the Commission recognizes that some research data cannot be made open and applies the principle of *'as open as possible, as closed as necessary'*. It is therefore possible to opt out of research data sharing at any stage - before or after the signature of the grant agreement - but reasons have to be given e.g. for intellectual property rights (IPR) concerns, privacy/data protection concerns, national security concern, if it would run against the main objective of the project or for other legitimate reasons (see General Annex L of the 2017 Work Programme adopted at 25 July 2016).

2. Data summary

The Impact Diabetes Bump2Baby (IDB2B) data management plan (DMP) aims to provide a strategy for managing data generated and collected during the project and optimise access to the and re-use of research data. The DMP is intended to be a ‘living’ document that will outline how the IDB2B research data will be handled

during and after the project. As such, it will be reviewed and updated at regular intervals (see section 6 for details)

The DMP describes the data management cycle for all datasets to be collected, processed and/or generated by the research team. It covers:

- data handling during and after the project
- types and formats of data will be generated/collected
- methods and standards applied
- whether data will be shared or made open-access and how
- the data curation and preservation

IDB2B will generate diverse outputs including measurement data, interview recordings, survey results, protocols and scientific articles.

2.1 Purpose of data collection/generation and relation to project objectives

The purpose is to collect and analyse data on the impact of IDB2B within a multicentre randomised trial. The data collection will comply with all national, EU and third country ethical and legal requirements. Access to use these data are needed to address the different IDB2B objectives as specified in the grant agreement and summarised below:

Overall objective:

To contribute to the early prevention of maternal and child diabetes, overweight and obesity and other non-communicable associated diseases (hypertension, cardiovascular disease, metabolic syndrome, cognitive deficits and behavioural issues).

Specific objectives:

System Co-Creation Objective: To combine evidence-based antenatal and postnatal lifestyle interventions programmes into a single, seamless support programme (IDB2B) that optimises health outcomes for the mother and baby and contributing to the prevention of T2DM and adverse long-term programming effects in the offspring.

This will be achieved through following sub-objectives:

1. To synthesise the lifestyle intervention literature, extract successful core components and those that can be modified in implementation and engage stakeholders across the different implementation context and situations to identify enablers, barriers and resource needs and co-design for IDB2B in Ireland, UK, Spain, and Australia. (WP1, WP3)
2. To co-produce the novel Health-e Mum, Health-e Baby app through stakeholder engagement, co-design, consolidation and optimisation of four evidence-based interventions and the broader evidence base into a single, sequential, state-of-the-art lifestyle behaviour change app that will coach a woman through the majority of her baby's first '1000 days' and that will be adaptable to different contexts and situations. (WP2)
3. To co-design an implementation toolkit to inform future implementation at the level of health services for sustainability and scalability, including mechanisms to build local capacity for sustained implementation of the programme following project completion. (WP1-8)

Scientific Objective: To evaluate the efficacy of IDB2B for the mother (maternal weight gain during pregnancy and weight loss post pregnancy) and baby (infant growth pattern, neurodevelopment and eating behaviour) and its contribution to reducing risk of transition to T2DM, overweight/obesity and other non-communicable diseases.

This will be achieved through following sub-objectives:

1. To undertake a randomised controlled trial (RCT) of 800 women (200 women per country)(WP5) and to evaluate its efficacy on achieving appropriate gestational weight gain (GWG) and reducing postnatal weight retention based on a difference in BMI of 0.8kg/m^2 between groups at 12-months postnatal. (WP6)
2. To examine the efficacy of IDB2B in promoting optimal infant growth pattern, neurodevelopment and

eating behaviour. (WP6)

Societal Impact Objective: To demonstrate the IDB2B's feasibility delivered across diverse health care systems and contexts.

This will be achieved through following sub-objectives:

1. To deliver the intervention across a range of settings and contexts capturing efficacy and quantitative and qualitative implementation data to inform future implementation and scale-up of IDB2B. (WP6)
2. To calculate the cost-effectiveness for potential health service savings of IDB2B implementation and compare the cost-utility across countries to evaluate the impact of context variation on cost-effectiveness. (WP6)
3. To develop a future rollout action plan for IDB2B, that will include the implementation toolkit and policy options, in the four target countries and wider EU audience to enable its sustainability and scale-up through engaging key decision makers and other stakeholders, enabling further co-design, raising awareness and improving dissemination throughout IDB2B. (WP8)

2.2 Types and formats of data generated/collected

Types of data:

- Self-administered online questionnaires:
- Clinical assessment: physiological, cognitive and anthropometric measures
- Measurements in biological tissue
- Molecular data: data on (part of/whole) genome, transcriptome, proteome, metabonome
- Modelling data: economic impact data
- Stakeholder personal information for the purposes of communication, dissemination and exploitation activity: For example stakeholder name, job title, company name, email address, contact telephone number, postal address, area of expertise.

Formats of the data:

- Data and metadata will requested, stored and transferred (across partners and in X) in comma-separated values (CSV) format.
- To facilitated the data exchange, MS Excel compatible files including comma separated and .xls(x) format will also be accepted.
- Stakeholder personal information will be stored electronically within .xls(x) format and .doc(x) documents. It is anticipated that paper formats will be utilised at events where undertaking an electronic storage will not be possible. This data will then be inputted electronically and the paper copies destroyed to comply with national and EU legislation.
- For statistical purposes, other formats include .sas7bdat (SAS), .Rdata (R), .SAV (SPSS)
- Where applicable data formats may be migrated when new technologies become available and are proved robust enough to ensure digital continuity and continues availability of data.

2.3 Specify if existing data is being re-used (if any)

WP1 Intervention Optimisation will collate a series of systematic reviews within pregnant and postpartum women. The WP use the data from these previously conducted reviews to construct the optimisation report (D1.1). The underpinning systematic reviews will have peer-review publications associated with them.

2.4 Specify the origin of the data

WP2-6 will generate original data that have resulted from the development and delivery of the IDB2B intervention within women at high risk of developing GDM within 4 separate countries (Ireland, the UK, Spain and Australia).

WP8 will obtain stakeholder personal information to ensure the effective communication, dissemination and exploitation of the project to parties and individuals interested. These will only be obtained when there is a legal basis to do so. Legal basis' to do so includes consent, legal compliance and legitimate interest. This is explored in more detail within the project websites privacy notice.

2.5 State the expected size of the data

The size of the data will be evaluated over the course of the project. The expected size will depend on the extent and nature of the data made available.

2.6 Outline the data utility: to whom will it be useful

- IDB2B consortium
- European Commission services and European Agencies
- EU National Bodies
- Broader scientific community
- General public

3. FAIR data

3.1 Making data findable, including provisions for metadata

3.1.1 *Outline the discoverability of data (metadata provision)*

A specific template has been defined in order to describe, discover and trace existing data collected by the IDB2B project and the data that will be generated over the project lifespan. The template will be sent and filled out by the data owners/providers and saved on the IDB2B repository. Afterwards a metadata fiche, for each IDB2B data collection, will be made publicly accessible through Zenodo (https://zenodo.org/). Zenodo is an open access, open source, open data platform that originated as a catch-all repository for EC funded research. The platform has since expanded to develop tools for big data management and extended digital library capabilities. Zenodo data and metadata will be retained for the lifetime of the repository. This is currently the lifetime of the host laboratory CERN, which currently has an experimental programme defined for the next 20 years at least.

In addition a list of studies and available variables will be compiled, which will help IDB2B data users to identify potential datasets for analysis.

3.1.2 *Outline the identifiability of data and refer to standard identification mechanism*

The DCMI metadata terms will be used for IDB2B project data (https://www.dublincore.org/specifications/dublin-core/dcmi-terms/). Each dataset is specified with the following minimal set of attributes:

Name:	A token appended to the URI of a DCMI namespace to create the URI of the term.
Label:	The human-readable label assigned to the term.
URI:	The Uniform Resource Identifier used to uniquely identify a term.
Definition:	A statement that represents the concept and essential nature of the term.
Type of Term:	The type of term: property, class, datatype, or vocabulary encoding scheme.

The assignment and management of persistent identifiers (PIDs) to the data will be conducted in the first year of the project. A DOI is issued to every published record on Zenodo. The DOI is a top-level and a mandatory field in the metadata of each record. Metadata of each record is indexed and searchable directly in Zenodo's search engine immediately after publishing. Metadata of each record is sent to DataCite servers during DOI registration and indexed there.

3.1.3 *Outline naming conventions used*

For metadata, dataset and template names, we will use 3 mandatory parts:

- A prefix, indicating if it is a dataset, metadata or a template
- A root composed of:
  - A short and meaningful name
  - An acronym/short name of the data provider organisation (IDB2B is the default for templates)
  - A suffix indicating the date of the last upload into the repository in DDMMYYYY format.

Each of the elements will be separated by an underscore symbol.

3.1.4 *Outline the approach towards search keyword*

The dataset information reported in the metadata fiche will be published in Zenodo, where specific filters based on the metadata elements, and will allow searches to be refined across datasets through the use of terms or keywords.

3.1.5 *Outline the approach for clear versioning*

The versioning management of the data, metadata template and in general the files stored into the repository will be applied at 2 levels:

1. Via the naming convention and the use of the date as suffix, indicating the last version of the the file uploaded into the repository.
2. Within the NUID UCD repository set up for the project.

3.1.6 Specify standards for metadata creation

Zenodo's metadata is compliant with DataCite's Metadata Schema minimum and recommended terms, with a few additional digital library and big data tool enrichments. The DCMI have set standards for metadata. They are detailed via the links below.

Properties of elements: contributor, coverage, creator, date, description, format, identifier, language, publisher, relation, rights, source, subject, title, type

3.2 Making data openly accessible

3.2.1 Specify which data will be made openly available

To share data with IDB2B consortium partners, a repository has been set up at NUID UCD. It will provide access to data through a secure server.

The IDB2B repository will:

- Facilitate storage and sharing of data, results and intermediate results
- Be hosted at NUID UCD with the server located in Dublin, Ireland.
- Enable data users to work with selected quality controlled data sets and versions approved by the data owners/providers
- Enables flexible and detailed data sharing
- Reach the highest level of GDPR compliance
- Rely on EU security protocols for data sharing
- Apply a strict policy in granting and revoking data access
- Logging user identity during data access, download and upload including version control.

Table 3.1 Expected levels of accessibility for IDB2B data

Dataset number	Task number	Dataset name	Open/ Restricted	Reason for restriction
1	1.1	Scoping systematic review	Open	
2	1.4	Integrated health economics analysis	Open	
3	2.1	App specification	Restricted	Sensitive
4	2.2	Content library	Restricted	Sensitive
5	2.3	Health coach manual and fidelity checklists	Restricted	Sensitive
6	3.1	Contextual mapping tool and protocol	Open	
7	3.2	Contextual mapping across sites	Restricted	GDPR
8	3.3	Normalization factors for GDM screening tool	Restricted	GDPR
9	4.1	GDM screening tool training and fidelity protocol development	Open	
10	4.2	Training and fidelity monitoring	Open	
11	4.3	Training effectiveness and cost assessment	Open	
12	5.1	RCT protocol	Open	
13	6.2	Data analysis plan	Open	
14	6.4	Budget impact analysis	Restricted	GDPR
15	6.5	RCT database	Restricted	GDPR

16	8.2-8.4	Stakeholder personal information	Restricted	GDPR

Protocol: Making IDB2B Data Openly Accessible

- To encourage re-use and futher application of project results, all IDB2B data that underlies scientific publications will be made available via open-access online platforms, unless subject to protection or if release of all or part of the data to open-access would jeopardise the action's main objective.
- Data that results from IDB2B activities that underlies scientific publications must be submitted to the relevant IDB2B Task Leader and database manager not more than 10 days following any related publication in scientific journals (unless data is subject to protection or embargo periods). An information template will be circulated to all beneficiaries upon publication of the DMP outlining the descriptive information required by the database manager to evaluate and approve datasets for upload to open-access repositories. Upon receipt of data, the project manager will evaluate each dataset and request additions or modifications in a timely manner, to facilitate upload of the dataset by task leaders no more than 30 days after the original date of publication. It is the responsibility of project partners to prepare the template for submission in a timely manner to facilitate this process.
- All data collection should be completed prior to the official deadline as outlined for each task at the end of the Grant Agreement. Partners are expected to observe such deadlines and have all data in a suitable format ready for sharing openly according to these deadlines unless the publications have not yet been accepted.
- Partners who intend to protect their data should notify all consortium beneficiaries, the project coordinator, and the database manager as soon as possible to ensure that the optimum level of confidentiality is upheld from an early stage. Evidence of applications for protection, and/ or associated legal processes, should be sent to the database manager within six months of such notifications. If no evidence of protection is provided, the IPC may request that such data be made accessible.
- When considering the potential to make data open access, Partners are requested to review the project Consortium Agreement which follows the standard rules as outlined in the DESCA model (http://www.desca-2020.eu/) for Horizon 2020. This defines the main approach regarding the ownership, protection and access to key knowledge like IPR and data. This approach will allow the IDB2B partners, collectively and individually, to pursue opportunities arising from the project's results.

3.2.2 Specify how the data will be made available

Zenodo will be used to store metadata, which will be openly available. Aggregated data and de-identified data are only directly integrated into Zenodo if the conditions are set by the data owner in the IDB2B repository are to allow this type of access. The data owner can indicate to grant/revoke access and user groups as specified in 2.6.

Zenodo metadata for individual records as well as record collections are harvestable using the OAI-PMH protocol by the record identifier and the collection name. Metadata is also retrievable through the public REST API.

3.2.3 Specify methods or software tools are needed to access the data

OAI-PMH and REST are open, free and universal protocols for information retrieval on the web. No specific methods or tools are needed to access the metadata. Metadata are publicly accessible and licensed under public domain. No authorization is ever necessary to retrieve it. In the case of specific software tools being used to generate data, these will be specified clearly with the associated dataset and metadata.

3.2.4 Specify where the data and associated metadata, documentation and code are deposited

Initial data will be deposited in the NUID UCD repository. The sharing of data will occur via Zenodo and associated data uploaded in appropriate formats with metadata.

3.2.5 Specify how access will be provided in case there are any restrictions

The IDB2B repository will have restricted access. Data sharing will occur with partners during the project. Only de-identified and checked data will be shared. Data access will be determined by the Project Management Team, with final decision located with the Project Coordinator. As the project progresses and data is identified and

collected, further information on making data openly accessible will be outlined in subsequent versions of the DMP. In specific, information on methods or software tools needed to access the data, information on where data and associated metadata, documentation and code are deposited and how access will be provided in case there are restrictions.

Upon completion of the project, data suitable for open access will be uploaded to Zenodo. Zenodo data and metadata will be retained for the lifetime of the repository. This is currently the lifetime of the host laboratory CERN, which currently has an experimental programme defined for the next 20 years at least. Metadata are stored in high-availability database servers at CERN, which are separate to the data itself. Other data within the full repository will be archived at NUID UCD for 25 years. Application to access data within the repository can be made to the Project Coordinator.

3.3 Making data interoperable

Partners will observe OpenAIRE guidelines for online interoperability, including OpenAIRE Guidelines for Literature Repositories, OpenAIRE Guidelines for Data Archives, OpenAIRE Guidelines for CRIS Managers based on CERIF-XML. These guidelines can be found at: https://guidelines.openaire.eu/en/latest/ . Partners will also ensure that IDB2B data observes FAIR data principles under H2020 open-access policy: http://ec.europa.eu/research/participants/data/ref/h2020/grants_manual/hi/oa_pilot/h2020-hi-oa-data-mgt_en.pdf

Information relating to the interoperability of IDB2B datasets has been collated in Annex 3: Data inventory table. As the project progresses and data is identified and collected, further information on making data interoperable is drafted in Annex 1. Further information on making data interoperable will be outlined in subsequent versions of the DMP. In specific, information on data and metadata vocabularies, standards or methodology to follow to facilitate interoperability and whether the project uses standard vocabulary for all data types present to allow interdisciplinary interoperability.

At present, no specific data and metadata vocabularies are available for the field of health interventions in pregnancy and postpartum. A common vocabulary is located at Dublin Core Metadata Initiative (DCMI), which the Dublin Core metadata originated. This is a well describe schema for describing metadata and it will be applied to this project’s data. The data formats will be aimed at allowing data exchange. Zenodo uses JSON Schema as internal representation of metadata and offers export to other popular formats such as Dublin Core or MARCXML. For certain terms Zenodo refers to open, external vocabularies, e.g.: license (Open Definition), funders (FundRef) and grants (OpenAIRE). Each referrenced external piece of metadata with the Zenodo files is qualified by a resolvable URL.

3.4 Increase data re-use

IDB2B is expected to produce a substantial volume of novel data and knowledge through experimental approaches that will be presented to the scientific community, industry, policy-makers and society at large through a carefully designed portfolio of dissemination actions. Datasets uploaded in the ZENODO repository will be freely accessible after an embargo period determined per dataset, if required. Potential users are expected to adhere with the ZENODO Terms of Use and will be subject to scrutiny by the ZENODO team.

As the project progresses and data is identified and collected, further information on increasing data re-use will be outlined in subsequent versions of the DMP. In specific, information on how data will be licenced to permit the widest reuse possible, when the data will be made available for re-use, whether the data produced and/or used in the project is useable by third parties, a description of data quality assurance processes and specifications of length of time for which the data will remain re-usable will be provided.

3.4.1 Specify how the data will be licenced to permit the widest reuse possible

The Zenodo platform will be used to publish the research data and includes an open definition license. Data downloaded by the users is subject to the license specified in the metadata by the uploader. Each record contains a minimum of DataCite's mandatory terms, with optionally additional DataCite recommended terms and Zenodo's enrichments. Metadata can optionally describe the original authors of the published work. Zenodo is not a domain-specific repository, yet through compliance with DataCite's Metadata Schema, metadata meets one of the broadest cross-domain standards available.

3.4.2 Specify when the data will be made available for re-use

Currently the periods of embargo that are planned relate to publication of data but all research data will be reviewed as the project progresses and where an embargo is deemed necessary, this will be documented and recorded.

3.4.3 Specify whether data produced and/or used in the project is useable by third parties

The de-identified data produced by the project will be useable by third parties after the end of the project. There may be some restrictions applied due to the nature of the data but these will be clearly documented.

3.4.5 Describe data quality assurance processes

All data and metadata uploaded to Zenodo is tracable to a registered Zenodo user. Each record contains a minimum of DataCite's mandatory terms, with optionally additional DataCite recommended terms and Zenodo's enrichments. IDB2B will have a dedicated data manager, who will be responsible for maintaining the overall IDB2B repository and associated Zenodo data sets. Standard operating protocols will be devised for all data collection. A data analysis plan will specify all protocols associated with data cleaning, analysis and storage.

3.4.6 Specify the length of time for which the data will remain re-usable

The lifetime of the laboratory CERN, which hosts Zenodo, currently has an experimental programme defined for the next 20 years at least. Metadata are stored in high-availability database servers at CERN, which are separate to the data itself. Other data within the full repository will be archived at NUID UCD for 25 years.

4. Allocation of resources

4.1 Estimated costs for making data FAIR

Costs for establishing and maintaining the IDB2B repository are covered by the financial budget of the IDB2B H2020 funding.

4.2 Clearly identify responsibilities for data management

The Project Management Team at NUID UCD will manage the IDB2B repository, with these specific responsibilities:

- Initial set-up of hardware and software components of the data repository
- Maintenance and capacity management of the data repository
- Carrying out the initial security assessment of the repository
- Performing security assessment on a regular, annual basis to guarantee the agreed security level
- Reporting and blocking any possible security threat, taking appropriate measures accordingly
- Creation and management of the internal user group account database (UGAP), as one of the components of the data repository
- Co-creation of the data repository's folders/sub-folders for each user group and document type (e.g. data, metadata, templates)
- Collecting the user requests for access to and download of data
- Preparing, checking the list of user groups and members for each user group
- Definition, creation, updating of the data repository structure i.e. folder and sub-folder structure, names, contents and access, upload and download permissions

NUID UCD is not responsible for:

- Interruptions in the data repository services that are due to force majeure.
- The content (of data and documents) reported into the data repository, that must be compliant with this DMP.

NUID UCD's obligations shall cover:

- Not distributing the list of members for each user group, except to IDB2B Steering Committee membership.
- Informing the IDB2B Steering Committee about any scheduled interruptions dut to data repository services upgrading or technical interventions at NUID UCD.
- In general terms comply with personal data protection rules (Regulation EC 45/2001) and GDPR 2016/679.

4.3 Describe costs and potential value of long term preservation

While the repository in itself is not maintained after the end of the project, all files stored within the repository shall be stored after the project to maintain the requirements of good scientific practice. The lifetime of the laboratory CERN, which hosts Zenodo, currently has an experimental programme defined for the next 20 years at least. Metadata are stored in high-availability database servers at CERN, which are separate to the data itself. Other data within the full repository will be archived at NUID UCD for 25 years. IDB2B has the potential to be of value as a longitudinal multicentre cohort that could be studied for health and dietary associations into the future.

5. Data security

5.1 Data confidentially and integrity

All research data underpinning publications will be made available for verification and re-use unless there are justified reasons for keeping specific datasets confidential. The main elements when considering confidentiality of datasets are:

- Protection of intellectual property regarding new processes, products and technologies where the data could be used to derive sensitive information that would impact the competitive advantage of the consortium or its members,
- Commercial agreements as part of the procurements of components or materials that might foresee the confidentiality of data,
- Personal data that might have been collected in the project where sharing them is not allowed by the national and European legislation.

The data confidentiality and integrity are implemented at various levels:

- Data at rest in the IDB2B repository will be protected against unauthorised access by means of password protection and secure server. Appropriate access levels will be granted by the creation of user groups.
- Data in transit will be secured by means of secure data transfer mechanisms such as encryption, secure mail and password protection.
- Data access is logged by NUID UCD via server data logs.
- Consortium partners will impose a strict policy on all emplyees, co-workers and staff around access to data. This policy will include, but is not limited to:
    - Allowing copies on local devices only during the processing of data with guaranteed erasure after being processed
    - Extending the access control policies to local copies
    - Contractual clauses
    - Agreement to terms and conditions before access is granted
    - Data will be de-identified when shared with partners
    - Lastly, awareness on data privacy and security will be supported by local processes such as provision of education and resources to ensure compliance.

5.2 Data availability

Business continuity and data availability are guaranteed by NUID UCD, with a recovery time objective of 7 days and a recovery point objective of 2 days. Moreover a DPIA (Data Privacy Impact Assessment) will be conducted as well as other obligations under GDPR.

6. Ethical aspects

IDB2B has a dedicated WP for ethics (WP9) to ensure the ethical requirements are met for all research undertaken in the project, including data management aspects, in compliance with H2020 ethical standards. All partners will assure that EU standards regarding ethics and data management are fulfilled. IDB2B partners must comply with the ethical principles (see GA, Article 34) and confidentiality (Article 36). The transfer of data on human subjects to the IDB2B repository is only considered when: informed consents, ethics approval and – when applicable -approval by local data protection authorities cover the purpose that the data are envisaged to be used within IDB2B and allow transfer of individual or aggregated data to the IDB2B repository. More details in relation to Ethics and security for data management can be found in the GA section 5.

7. Other

The first version of the DMP will be validated by the consortium and will function as the operational plan until it is updated. The DMP will be updated over the course of the project whenever significant changes arise, such as:

- New data
- Changes in consortium policies
- Changes in consortium composition and external factors

The DMP will also be reviewed and revised as needed at 18-month intervals by the full consortium. Revisions will be documented in the version control table at the start of this DMP.

Annex 1 Data inventory tables

Summary

Dataset No.	Task No.	Dataset name	Data subset	Type of data	New/existing data	Method of data capture	Format of data capture	Expected size	Quality control procedures	Potential data user	Ethical issues? Y/N	Type of access (open/restricted)

Metadata provision

Dataset No.	Task No.	Data format	Metadata standard	Type of metadata associated with it	Metadata vocabularies	How will data be findable?	Any specialised software required to use data?

Storage of data

Dataset No.	Task No.	Dataset name	Data storage location (short-term)	Storage media	Data security provisions	Expected size of dataset	Length of time data will be stored	Data storage location (long-term)	Person responsible	Cost

Re-use of data

Dataset No.	Task No.	Dataset name	How will data be re-used?	Open/ restricted	Type of IP/ protection sought	Type of licensing agreement to be implemented

This project has received funding from the European Union's Horizon 2020 research and innovation programme under grant agreement No 874984.
